# Bacteriological research and ‘puerperal’ fever: female health and childbirth in late colonial India

**DOI:** 10.1017/mdh.2025.10020

**Published:** 2025-07

**Authors:** Kaushalya Bajpayee

**Affiliations:** Jindal Global Law School, O.P. Jindal Global University, Sonipat, India

**Keywords:** Puerperal fever, Bacteriology, Tropicality, Sanitation, Hygiene, Midwifery

## Abstract

This article explores female healthcare at the crossroads of bacteriology and obstetric research. Puerperal fever or childbed fever manifested as an epidemic since the nineteenth century, and in both Europe and America, it charted a distinct course for bacteriological research. With the identification of bacteriological causes, new sets of public health regimes were instituted in both regions. The experience of the colonies, however, differed. This paper focusses on how colonial discourse on obstetric nursing, midwifery, clinical hygiene, and maternal healthcare can be positioned in this global history of female health research. The paper explores why, in India, on one hand, bacteriological research in female health suffered in terms of priority (unlike that of cholera and plague) despite the alarming rate of maternal mortality. On the other hand, medical practitioners trained in Europe worked as the conduit through which the bacteriological research of Europe made its way into India. Contemporary documents reveal how colonial prerogatives were channeled through the race theories linked to Indian cultural practices related to midwifery and obstetric nursing, and how the female health discourse was still marred by the notion of tropicality.

## Introduction

This article explores female healthcare at the crossroads of bacteriology and obstetric research in India. Engaging with the trope of tropicality and its relation to bacteriological research in India, this article sheds light on colonial policies concerning female healthcare in the late nineteenth and early twentieth centuries. It demonstrates how theoretical frames of tropicality and racist theories went hand in hand to shape colonial policies on female health, particularly in the institutionalisation of childbirth and midwifery. Debates concerning puerperal fever (PF) were particularly significant, as in India as well as globally, it was a major driver of maternal mortality. These debates, it shall be argued, were enmeshed with ideas of race and tropicality and helped to shape policies on childbirth, nursing, and midwifery. Such policies, I shall argue, were a part of ‘moral prophylaxis’ shaped through the colonial ‘negative characterisation of tropics interwoven with wider racially defined cultural categories.’^1^

This paper focusses on the colonial discourses on female health in the context of bacteriological research and on how colonial policies were framed, reiterating ideas on tropical and racial subordination. It argues that germ theories were recentred towards a broader debate concerning cultural difference, which led to the formulation of a colonial policy framework galvanised around cultural theories associated with tropical childbirth practices. These, in no uncertain terms, were directed towards traditional midwives – *dhais* – and their lack of medical knowledge as well as their negligence of hygiene and sanitation, which hinted towards a ‘pathologisation of space’ particular to the tropics. Pratik Chakraborty argued that in the late colonial period, the ‘dual framework of moral geography and racial supremacy by incorporating the ideas of race and climate, conceptually and methodologically redefined bacteriology in the tropics.’^2^ This article analyses this redefinition of bacteriology through the lens of colonial policies towards maternal health in colonial India. In the British colonies, sanitation was often emphasised over quarantine in the response, for example, to outbreaks of plague and cholera.^3^ In many ways, this reflected wider global movements concerning public health which emphasized hygiene and sanitation as priorities.^4^ In the sphere of maternal health, these concerns were manifest in colonial authorities’ attempts to professionalise and institutionalise midwifery and obstetric nursing.^5^ Engaging with the immensely rich existing scholarship, this paper demonstrates how bacteriological research in female health (or rather the lack of it) had a direct bearing on the colonial health policies, especially in nursing and midwifery in the early twentieth century.

## Puerperal/childbed fever and bacteriological research

“Puerperal fever” emerged in European medical treatises and scientific texts as a distinct term in the eighteenth century. By this time, it was distinguished from after-pains, milk fevers, “military fever” (typhoid), and other fevers, and had attracted the attention of a range of medical theorists and scholars.^6^ By the late nineteenth century, more was written about puerperal fever as a cause of maternal mortality than any other disease; indeed, it was the most common cause of maternal mortality, killing an estimated quarter to half a million women in England and Wales alone during the eighteenth and nineteenth centuries.^7^

From the late eighteenth century, a number of theorists like Oliver Wendell Holmes (1809–1894) and Ignas Semmelweis (1818–1865) debated its aetiology, particularly, its spread and contagiousness.^8^ Scottish obstetrician Alexander Gordon (1752–1799) demonstrated in his 1795 *A Treatise on the Epidemic Puerperal Fever of Aberdeen* that puerperal fever could be contagious, and that birth attendants could transfer the infection between patients.^9^ A debate ensued concerning whether such deaths were more common in institutional deliveries or in home confinements. Gordon, and other later theorists such as Ignaz Semmelweis, argued that male midwives and physician-accoucheurs who handled more cases and who also saw other patients tended to have a disastrous effect on the spread of the infection.^10^ The implications of these findings were strongly opposed in many quarters and resisted by staff at many lying-in hospitals.^11^

By the late nineteenth century, bacteriology became the new science, with microorganisms moving into the realm of medicine. German physician, Robert Koch (1843–1910), identified several bacteria as causes of infectious diseases. It led to greater authority for experimental laboratory methods in medicine and was followed by a new discourse on immunology. It also led to the appreciation of a possible bacteriological basis for puerperal infections. In the first decades of the twentieth century, researchers in a number of countries worked to identify the particular organisms responsible for puerperal fever. Irvine Loudon argues that though the identification of *Streptococcus pyogenes* as a specific causal agent is attributed to Louis Pasteur, it really belonged to Amedee Doleris in 1880.^12^ In 1919, J.H. Brown, at the Rockefeller Institute for Medical Research, identified the beta hemolytic forms of the streptococci and the subsequent classification was developed by Rebecca Lancefield at the same institution in the 1930s. Later, Dora Colebrook and Rebecca Lancefield came to the conclusion in the 1930s that the Group A beta hemolytic streptococcus was the causative organism for human disease including that of puerperal or childbed fever. In 1935, Group B Streptococcus (Streptococcus agalactiae; GBS) was first identified as a cause of puerperal sepsis by Lancefield and Hare.^13^ But it was in the 1970s that American physicians were alerted to the increasing incidence of Group B beta hemolytic streptococcus (GBS) as a venereally transmitted infectious disease producing serious, and often fatal, neonatal morbidity.^14^

**Figure 1.**
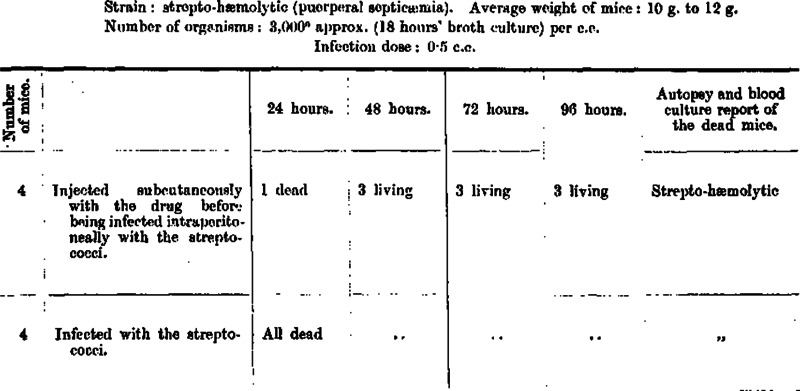
*Indian Journal of Medical Research*, October 1937, p. 467.

The *Indian Journal of Medical Research* reported their research conducted on the mice in the late 1930s, on haemolytic streptococcus (puerperal septicaemia) which was one of the most common causes of maternal mortality in the 1920s and 30s. The bacterial causes of such puerperal diseases were well established, though the government remained silent in terms of female healthcare policies in this regard (Figure 1).

Apart from puerperal fevers caused by streptococcal infection, puerperal sepsis was a frequent complication after childbirth. Tetanus was rare puerperal disease but was regarded as one of the gravest dangers of childbirth. In 1937, Ronald Hare, practising in Canada, argued that while puerperal sepsis was mostly contracted from outside, tetanus puerperalis was endogenous, generally contracted from septic procedures during delivery. The postpartum uterus was a good culture medium for the anaerobic organism, *Clostridium tetani*. So he believed that rather than altering the diet, it was the application of newer obstetric techniques which could prevent puerperal infection.^15^ The mysteries that shrouded the pathology of puerperal diseases, regarding their causation and spread, were cleared with the increasing acceptance of bacteriological discoveries.

### Bacteriological research and maternal health in India

Scholars have shown that bacteriological research in India was mainly focused on epidemic diseases like cholera and plague, and there was a serious lacuna in research into female health in particular.^16^ Pratik Chakraborty, in his study on ethics and the use of animal resources in scientific experimentation, discussed the politics of bacteriological institutionalisation in colonial India. He argued that science came to India as a positive moral force and though the bacteriologists were against the anti-vivisectionists, the British were able to secure immunity from an otherwise explosive political situation by assuming moral and political agency.^17^ In his memoirs, Ronald Ross (1857–1932) recalled that medical services were poorly organised and individual medical men were oblivious to medical advancements. Though they were engaged in good clinical work, they were unaware of the greatest bacteriological discoveries by scientists such as Robert Koch (1843–1910) and Louis Pasteur (1822–1895).^18^ Serious endeavours in this regard started only with the outbreak of plague in India in 1896–97,^19^ which changed the landscape for bureaucratic as well as societal response. David Arnold reiterates this point by highlighting the importance of contemporaneous discovery of the causes and means of communication of both malaria and the plague to energise both laboratory-based science and field research.^20^ However, there was hardly any discussion about the linkages between bacteriological work with female reproductive diseases in the contemporary medical literature. This is evident from the words of contemporary practitioners like Scottish doctor Margaret Balfour (1866–1945), who discussed the neglect of female health by colonial authorities in India, underscoring that the focus of the colonial administrators remained on the epidemics. ‘Control of such epidemics,’ she wrote, ‘is one of the major duties of district medical officers of health, and they also account for a great part of the expenditure on this branch of medical work. In the face of this need for control of diseases such as malaria, plague, dysentery and kala azar, it is small wonder that the problems of infant mortality have escaped the attention they deserve in India.’^21^ She further added that the lack of ‘voluntary effort’ and the problem of an ‘uninformed public about the true purpose of the work’ also prevented much headway in this regard. Recent scholars like Radhika Ramasubban maintain that medical research in the twentieth century remained focused on the needs of the segregated sector of the Army and the European civil population.^22^ Similarly, in her study on Bombay Presidency, Mridula Ramanna has shown that from the middle of the nineteenth century, the role of the government, in terms of research, was limited, and it was mainly due to contributions of philanthropists that maternal and infant health welfare was institutionalised.^23^ This was primarily because improving the health of women did not, in David Arnold’s words, ‘make the empire more efficient and profitable’.^24^ Colonial considerations prevented the development of female healthcare in the same way that colonial administration handled epidemics like plague and cholera, especially because of the effect of cholera and plague on the European population and the British Army.^25^ This lacuna was voiced by medical officials as well. For instance, Dr J. M. Orkney, the director of Maternity and Child Welfare in the All India Institute of Hygiene and Public Health, Calcutta, expressed his dismay, arguing that the cholera problem was taken seriously and was tackled by the government in all earnestness, though it neither left behind a disablement nor was its effect on the future generation comparable with the ravages caused in the family by the death or ill health of the mother. But, he complained, very little was done for the protection of mothers and children.

In 1904, Curzon mentioned in unequivocal terms: ‘In our overwhelming work and admitted lack of technical knowledge, we bow down to the man of science … When we do not take their advice, it is only because administrative consideration of vastly greater importance, as in the case of plague, has a superior claim.’^26^

Besides, education and research were generally kept apart. Dr Bradfield, a member of the Indian Medical Service (IMS), argued while presenting a paper before the South Indian Branch of the Indian Medical Association, that though the research institutes were doing ‘important work’, they did no ‘original’ research.^27^ This lack of original bacteriological research in India was ascribed to a kind of ‘stasis’. Sheldon Watts, in discussing the British response to cholera in India and Cairo, argued that the policy on cholera since 1868 marked a reversal of British research policies. Contradicting the findings of the Cholera Conferences,^28^ the British medical scientists and administrators opined that Indian cholera was not contagious and not caused by a germ, thus denying the efficacy of quarantine and deterring scientists from further work on germ theory.^29^

As Deepak Kumar has argued, racial over tones were predominant in the colonial understanding of native capabilities to undertake scientific activities: ‘original productivity could not have struck deep roots in India, in the midst of racial discrimination.’^30^ Well into the twentieth century, such discriminatory tones were reiterated: Austen Chamberlain (1863–1937), Secretary of State for India, in his speech at the Indian section of the Royal Society of Arts argued that ‘For the task of research and alleviation, this country should always send its best to India‥ appealing to the leaders of medical profession to make themselves aware of the openings which service in India afforded in practice and in research.’^31^

However, with the coming of the germ theories and the identification of bacteriological causes for PF, female health issues were a continuing matter of concern in India, especially in view of rising maternal mortality and infant mortality rates in the 1920s and 30s.^32^ David Arnold identified a shift in terms of medical observation and research from the 1920s to the ‘hitherto neglected locations as the village, factory, school and women’s hospital and to new, or previously ignored, diseases.’^33^

## Tropicality and maternal health: rule of colonial difference

In this context, the notion of ‘tropicality’ continued to define ideas in colonial female health discourse. In the eighteenth century, the idea of tropics, as hot, dusty and unhealthy sites, identified as ‘real hot-bedshot-beds of diseases’ and tropical afflictions,^34^ which were often considered ‘no place for white men.’^35^ However, the paradox of the “white man’s burden” in such tropical areas was resolved by, as argued by Warwick Anderson, the study of *acclimatisation –* ‘a potent brew of race theory, geographical pathology and global politics.’^36^ For the Europeans in the colonies, the *failure of acclimatisation*, Anderson argues, eventually led to a buttressed conviction that ‘sanitary barriers could deflect portable tropical pathogens away from still vulnerable aliens.’^37^ For the Indians, it resulted in the discourse of ‘difference’.

The idea of tropics evolved in the European perception from a land unknown to an exotic place to a part of the savage, irrational and uncivilised ‘other’ of the Orient.^38^ In the eighteenth century, the linkages between tropical heat and putrefaction were reiterated through the concept of ‘miasma’. Miasma theory was one of the earliest tangible theories used to explain the spread of contagious diseases. It held that diseases spread by the stench of decay or putrefaction. The air was poisoned through such decay and was considered a cause of disease spread.^39^ However, with the territorial expansion of the British, the adaptability of British and Indian bodies to the geographical specification of the tropics became a cause of concern, ultimately resulting in the theorising of categorical racial theories justifying the hegemonic existence of the rulers in the colonies. These theories and the deadly scourge of diseases like Asiatic cholera and plague in the late nineteenth century entrenched the idea of ‘unhygienic, unhealthy and unclean colonies with their social and cultural backwardness.’^40^

A new medical specialisation investigated the tropical diseases, and as Chakraborty has pointed out, ‘from the late nineteenth century, the country experienced sustained bacteriological institutionalisation. Following the discoveries of Louis Pasteur and Robert Koch, bacteriology appeared as a panacea against the local scourge in colonial India.’^41^ In the colonies, the ideas of Pasteur and Koch’s germ theory and vaccines were subsumed within the colonial ideas of the tropics in creating links between germs, climate, culture and race, in the process leading to an abstruse metamorphosis of bacteriology in colonial India. Particularly in the discipline of obstetrics and gynaecology, this created a new discourse on hygiene and sanitation that was intimately associated with the pathogenic causes of diseases. While the colonial imperatives of economic and social policy-making dictated the terms of bacteriological and aetiological research into diseases like cholera and plague, female health issues and morbidity associated with PF failed to garner government attention. Rather, ideas of tropicality and tropical theories of hygienic backwardness fitted well with ideas of colonial racial supremacy.

Since the late nineteenth century, the idea of tropics defined medical ideas on colonial climate, bodies and cultural practices and there was a marked shift in medical research with the coming of germ theories. These replaced miasma theory – ‘an earlier environmental paradigm of disease which stressed the role of climate, topography, vegetation and soils, and also some aspects of social and cultural environment, in the aetiology and transmission of epidemic diseases.’^42^ But, here I argue that in the case of female health, the germ theories related to PF were appropriated through ideas of tropicality and miasmatic ideas of uncleanliness, lack of hygiene and putrefaction. As Chakraborty argued, “Imperial attitudes towards the tropics and a Victorian moralistic distaste for tropical pathogens shaped Pasteur Institutes and bacteriological practices in India. Nineteenth and twentieth century medical discourse defined climate in the tropics through moralistic idioms.”^43^ Understandings of tropicality also shifted in emphasis from the climate to racial and cultural peculiarities and tropicality’s impact on bodies. As argued by Partha Chatterjee, the ‘rule of colonial difference’ was based on the racial hierarchy of the Europeans in respect to the ‘incorrigibly inferior’ colonised (by virtue of their biology). He argued that “the European criticism of Indian tradition as barbaric had focused to a large extent on religious beliefs and practices.”^44^ The most important evidence of this was the ‘zenana’ and how the culture of confinement to the *zenana* impacted the bodies/health of the tropical/Indian females.^45^ Colonial obsession with the unpenetrated and impenetrable *zenana* reiterated the racial trope of ‘backward, uncivilised and incapable Indians’.^46^ The beginning of institutional intervention in female health, through the Dufferin Fund,^47^ was guided by the “white woman’s burden” of doing something ‘for the poor creatures.’^48^ Apart from policies, the notion of colonial difference was also evident in ideas on colonial anatomy. The constitutional differences of the colonial bodies from their European or American counterparts were often highlighted in diagnosis and medication. In 1932, the *Indian Medical Gazette* reported on the use of mercurochrome for puerperal cases as: “It is a matter of common knowledge that generally what is recommended as a normal dosage for Europeans or Americans is found to be much more than what is suitable for Indians. This is because of the less robust constitution of Indians.”^49^

## Puerperal fever, maternal mortality and women in the Tropics

Medical journals like the *British Medical Journal* and the *Indian Medical Gazette (IMG)* reported that a large number of women succumbed to postpartum fevers, often occurring three to four days after childbirth. While in England the number was negligible, ‘in Calcutta it accounted for 856 of the registered deaths (1907) of which no less than 723 were in babies under three months old.’^52^ Most of the time, the women were healthy, and the death was a sudden occurrence, which hardly provided any time for medical intervention. Cases of PFs showed sudden symptoms of fever, eclampsia and seizure. Dr C. Barry, Civil surgeon in Maymyo (Burma), reported in *IMG* about the case of a pregnant woman who was admitted with intense headache, neck pain and stiffness and a temperature of 103 degrees. Later, she was seized with an epileptic convulsion followed by a badly bitten, swollen tongue, hurried and shallow respiration and a feeble pulse.^53^ During delivery, women were also affected by puerperal mania and eclampsia.

The hospitals regularly reported on the obstetric cases and gave a detailed account of maternal mortality occurring in the prenatal and postnatal days. In 1926–1927, Carmichael College reported that 316 patients were treated in the Obstetric Ward, including the maternity hospital. The report mentioned anaemia, eclampsia, puerperal septicaemia and pneumonia as major causes of maternal mortality (Table 1).

**Table 1.** During 1926 -27, Carmichael College reported that 316 patients were treated in the Obstetric Ward, including the maternity hospital^50^

Anaemia of pregnancy	7
Eclampsia	4
Puerperal septicaemia	1
Pneumonia	1
Malaria Cachexia	1
Complications during puerperium Pneumonia – 1 Sepsis – 1	This was manipulated by ‘dhai’^51^ before admission, the patient died in hospital

**Figure 2.**
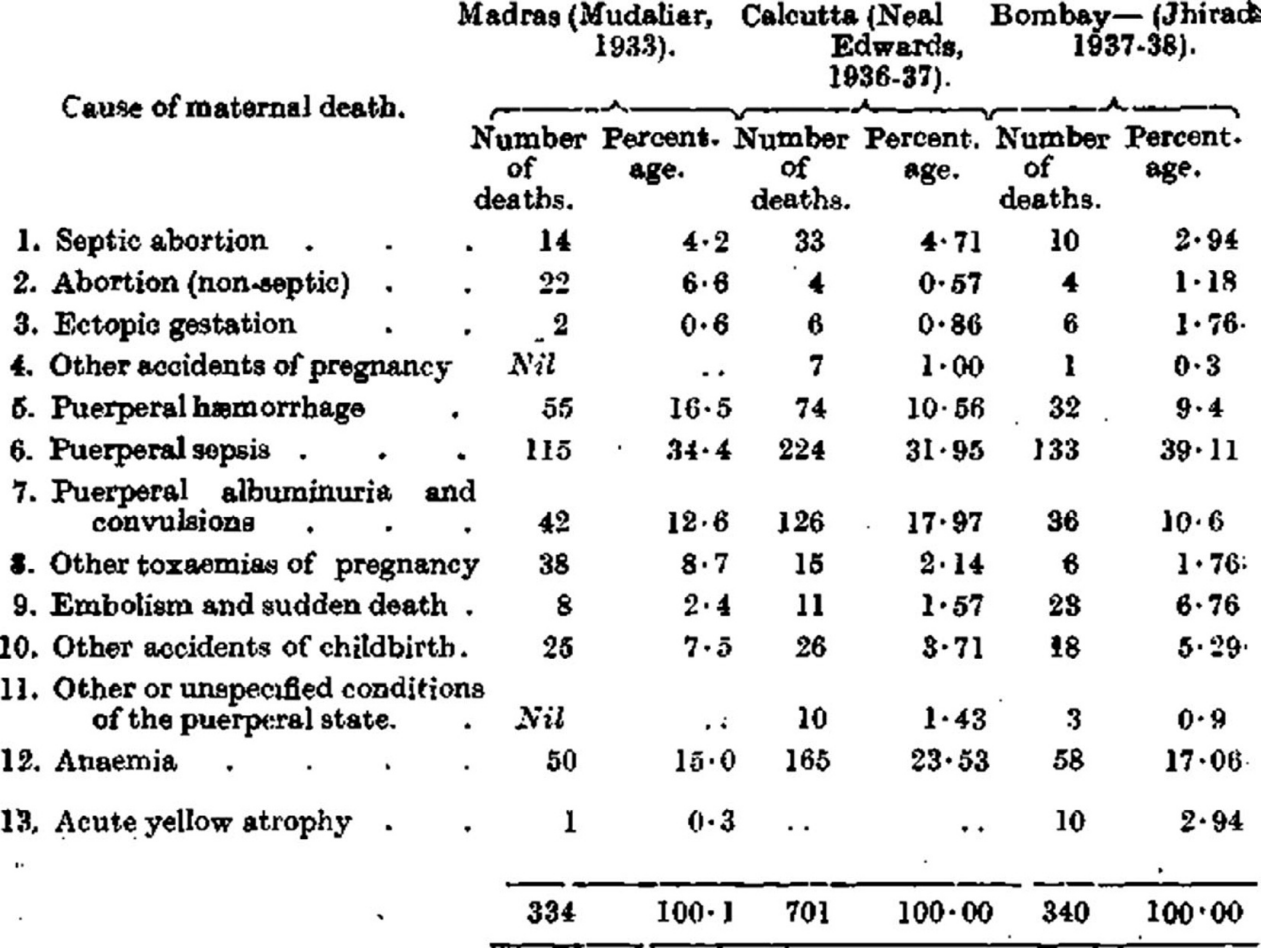
Report on an investigation into the causes of maternal mortality in the cities of Bombay, Calcutta, and Madras, 1937–1938, p. 2.

Anaemia of pregnancy was also a major cause of concern for women. In 1928, the *Indian Journal of Medical Research* reported on the existence of severe anaemia, and based on the reports of Balfour and other medics, this was most rampant among pregnant ladies in India.^54^ In 1932, the *IMG* reported that ‘tropical anaemia of pregnancy’ was different from anaemia of pregnancy or the pernicious anaemia of pregnancy. The distribution of the disease is most peculiar in that it is apparently extremely common in the tropics and only very exceptionally occurs in the temperate zones. In the following two tables, it is evident that maternal mortality in the 1920s and 1930s in the tropics was higher than in the temperate areas and that puerperal haemorrhage, puerperal sepsis and anaemia accounted for the highest number of maternal deaths in all three cities of Bombay, Calcutta and Madras (Figure 2; Table 2).

**Table 2.** Data collated from the *Indian Medical Gazette*, August 1932, p.402

Year	Europe	Cases of anaemia of pregnancy	Year	India	Cases of anaemia of pregnancy
1890 – 1920	Vienna Maternity Clinic	8	1925–1927	Bombay Hospital	125
1921 –23	Rotunda Hospital, Dublin	1	1926–1930	Seva Sadan Women’s Hospital, Calcutta	86
1923	Queen Charlotte’s Hospital, London	1	1928	Baroda State Hospital	4
1923	Simpson Memorial Hospital, Edinburgh	0	1928–1929	Cama and Albless, and Wadia Hospital	66

The susceptibility of tropical bodies towards puerperal diseases has been discussed in detail by Prof. V.B. Green-Armytage in his book *A Textbook of Midwifery in the Tropics.* Here, he identified PF in childbirth as a major contributor to the discourse on sepsis. In the foreword, Green-Armytage makes it clear how the gynaecological diagnosis and treatment was ‘different’ from in the West: ‘‥no author, graduate or doctor relying on western textbooks alone can appreciate how profoundly every problem of diagnosis and treatment is altered unless he has done an intern’s post in a women’s hospital, East of Suez.’^55^ Echoing the data given in the journals like *IMG*, he argued that complications were more acute for women in the tropics: Unlike Europe, he wrote, ‘In the tropics, the average maternal mortality is nearly 4 per cent and the incidence of complication over 20 percent. These high figures are due to lack of care, lack of diagnosis and lack of skilled treatment in a hot climate where the incidence of tropical disease alone is high and the general resistance to disease is low. For instance the average hemoglobin content of all European women is 80 percent and that of Indian women is 70 percent’.^56^ A common cause of fever in the tropics, associated with pregnancy as discussed by Green-Armytage, was pyelitis of pregnancy, usually occurring in the fourth month of pregnancy. Death in such cases was mostly due to pyaemia or septicaemia. The majority of infant mortality cases were ascribed to toxaemic conditions or syphilis of the mother, which also, he argued, could be very well prevented through antenatal treatment.

Green-Armytage observed that maternal mortality was high among the Hindus of Calcutta and the Muslims of Bombay, most of whom were ‘poor, inadequately nourished and lived in unhygienic surroundings’.^57^ He argued that most of the labour-related complications could be avoided through antenatal care, as part of preventive medicine. He prescribed 5 gms. of quinine to be taken daily for the last six weeks of pregnancy, both to lessen the chance of fever before and after childbirth and also to improve contractability of the uterus, thereby lessening the risks of tropical uterine inertia.^58^ Regular antenatal examination of pregnancy history (previous pregnancy, labour and puerperium) and tests like pelvimetry in the early stages were strongly advised with abdominal examination, vaginal examination and determination of relation between foetal head and pelvic brim in the later stages. Discussion on pelvimetry or pelvic index, since the late nineteenth century, as the measure of comparative evolution of races, asserted the racial hierarchy of European, Eurasian and Indian female bodies. The supposed disproportionality of the foetal skulls and the maternal pelvis in the Indian women was ascribed to the ‘unnatural’ life of the Indian women in the *zenana* which was cut off from light and fresh air.^59^

In the late 1920s, female reproductive health was drawn into the vortex of the controversy generated by the Child Marriage Restraint Act. The body of the child bride and the standardization of her age was the primary focus of the legislation. Recent works on the Act by historians like Ishita Pande, Ashwini Tambe and others have identified how such standardization ignored its impact on the child’s reproductive health. Rather the child served as a ‘site of sexual modernisation’ within the modernising agenda of roaring anti-colonial nationalism.^60^ In the context of this Act, Green-Armytage argued that marriage and conception at a lower age was not the main cause of infant/maternal mortality; rather, it was the lack of education of the midwives and the absence/ignorance of antenatal and post-natal care that led to the increased mortality rates. He identified the main causes of maternal mortality to be puerperal sepsis, toxaemias of pregnancy and complications in labour in the form of shock, haemorrhage or sepsis, which (except haemorrhage) could be prevented through proper antenatal and postnatal care and maintenance of hygiene and cleanliness.^61^ Green-Armytage identified postnatal care to be extremely important to prevent some symptoms which could otherwise arise. ‘Laceration of the cervix followed by infection and erosion is present in twenty percent of cases after the first baby,’ he wrote. ‘Immediate treatment with ten percent silver nitrate or electric cautery will cure most. In others, operative repair would be necessary. These lacerations and erosions may predispose to one-child sterility and later cancer.’^62^

As a part of the postnatal care, he also mentioned diets, suited to the Indian female physiology, which were distinctly different from the European diet charts. For the Indian females, he prescribed leafy vegetables (*sag*/*sak*) as part of the vegetarian diet and the mud fish like *koi*, *magur*, *singee* as part of the non-vegetarian diet. He included cereals like *Dhenki* (home-pounded) rice: *muri*, *khoi*, *chira*; *atta* and *suji*, while for the European diet, oatmeal porridge, brown bread, rusks and biscuits were advised.^63^

He also emphasised the role of birth attendants in dealing with such complications. ‘Even in cases of haemorrhage, prognosis depends on the promptness of treatment; post-partum haemorrhage can be prevented to a great extent by wise conduction of labour.’^64^ Thus, the discussion on prenatal and antenatal care to prevent bacteriological infection in puerperium redefined the role of childbirth practices and birth attendants. This was entwined in an imperial narrative of *disease, filth and putrefaction.* ^65^

The rise of maternal and infant mortality was linked by Green-Armytage to the spread of venereal diseases such as syphilis and gonorrhea in the tropics. Two per cent of foetal deaths were caused by premature interruption of pregnancy by syphilis. But it was argued that ‘In every suspected case of syphilis, Wassermann reaction of the mother should be tested and if positive, anti-syphilitic treatment carried out. Though the child could remain unaffected if the treatment was started four months before the birth of the child, a syphilitic child must be suckled with impunity by its mother, and it must not be given to a wet nurse.’^66^ In case of gonorrhoea, while the child was threatened with blindness, it made the mothers liable to puerperal infection and acute gonococcal septicaemia. Armytage also observed that gonococcal puerperal sepsis was not uncommon in the tropics, and the symptoms of fever subsided with regular care.

**Figure 3.**
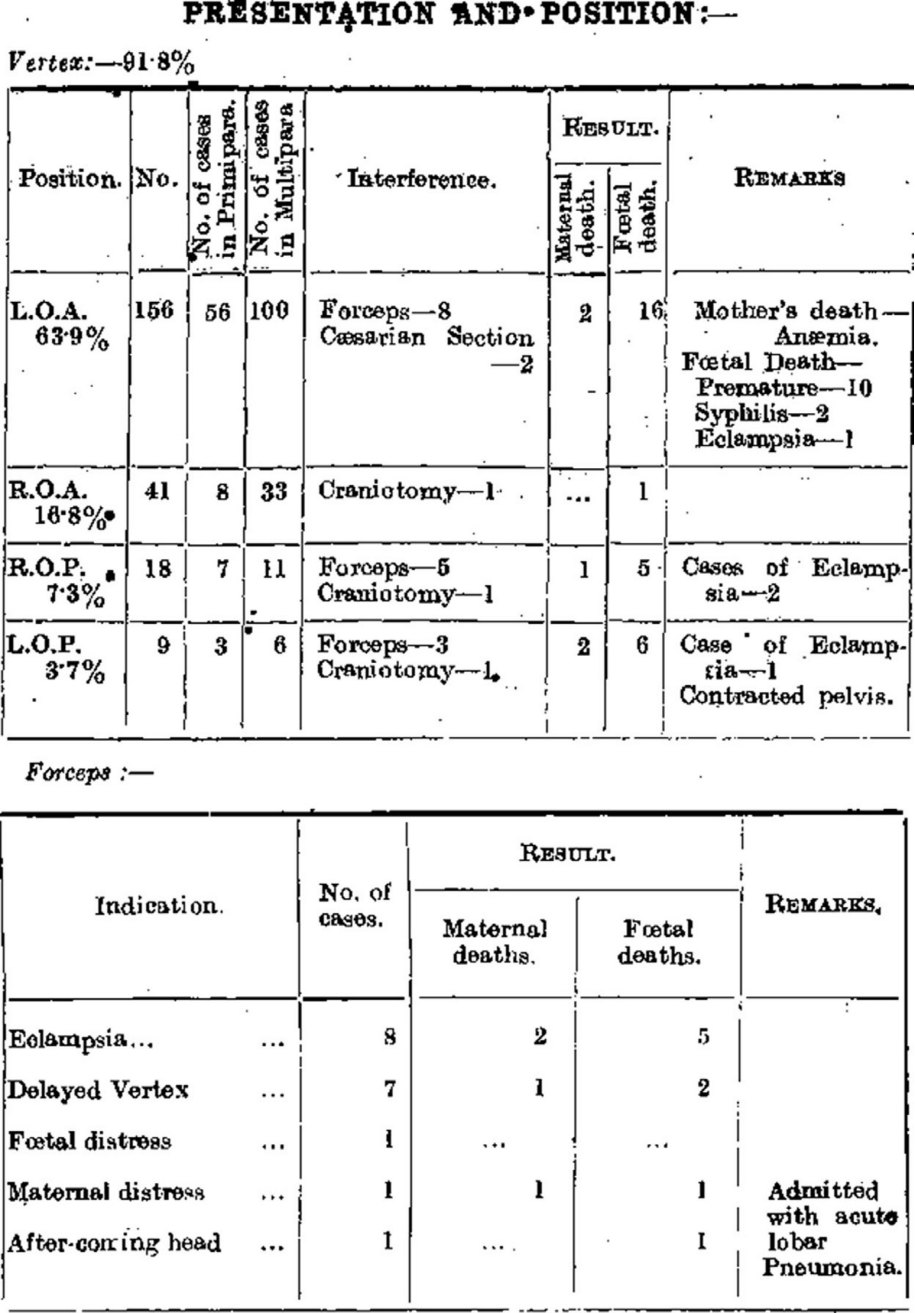
Complications and the causes of maternal death in the obstetric department in the Carmichael College and Hospital Report, 1926–1927.^73^

Like Green-Armytage, several Indian practitioners were reporting on the growing menace of puerperal diseases and bacteriological infections that affected maternal health. In 1927, A.L. Mudaliar and C.A.F. Hingston (IMS) identified that antistreptococcic serum in the case of puerperal sepsis was not as useful as had been anticipated, but it was significant as a prophylactic treatment. But they mentioned, ‘We have recently isolated a large number of different strains of streptococci – haemolytic and non – haemolytic – as well as some other organisms, and a polyvalent vaccine has been prepared and is being given a trial.’^67^ Kedarnath Das, the head of the midwifery department in the Campbell Hospital, regularly wrote columns on the medical advances made in the subjects of obstetrics and gynaecology. In his column on the existing medical literature, he mentioned ‘gas sepsis’, which was coined by William Welch in 1891 to designate an important group of fatal puerperal cases. Quoting from the *Johns Hopkins Bulletin*, he reported on the cases of gas sepsis where gas bubbles were found in the heart and blood vessels during autopsies.^68^ He identified that in these cases, where patients succumbed to the puerperal sepsis, ‘gas – basilli and gas passed from the uterus into the blood circulation during life.’^69^ He observed that in most of the cases, death was sudden, and there had been some kind of operative interference preceding the infection. Dr Das, in 1923, modified the forceps to suit the anatomy of Bengali women. He argued that because of early childbirth in India, the British forceps manufactured to British standards caused injuries to Bengali mothers.^70^ The following figure gives detailed data on the injuries caused by the use of forceps and during Cesarian operations (Figure 3).

Tuberculosis was another disease of concern affecting pregnancy. In pregnancy, the disease progressed very rapidly, aggravating the puerperal condition, often associated with repeated evening fevers, cough and copious sputum. In seventy per cent of cases, the child, after birth, living with the same condition, got infected with the disease. As Supriya Guha has argued, race and class were intimately connected with the discussion of tropical cultural tropes.^71^ Tuberculosis and toxaemias of pregnancy were said to be closely related to the custom of seclusion, and their greater occurrence among Muslim women was cited as proof.^72^

In all these cases, it was repeatedly reported that obstetric care was crucial in preventing these occurrences. In view of the large number of maternal and infant mortality in the tropics, medical practitioners unequivocally argued for a new discourse on hygiene and care to be introduced into the hospital schedule for prenatal and antenatal confinements. The tropicality of the diseases, as well as the physiology of the patients, were identified as major causes of such higher morbidity.

Ideas on ‘tropicality’ were not only restricted to parturient bodies but also to the practices associated with childbirth. While discussion on childbirth practices has been taken up in much detail by medical historians, this paper shall focusses on linkages to bacteriological research and how this had a direct bearing on colonial policies. In Europe, role of bacteria in puerperal fever as a major cause of maternal mortality was an established fact. These ideas were making their way to the colonies through the western practitioners. However, medical practitioners in India, in the absence of bacteriological research, focused on the vulnerability of tropical bodies and the backwardness of tropical childbirth customs. The following two sections, demonstrate how these ideas influenced colonial policies and how they were politically justified in the globally emergent modern language of sanitation and hygiene that *needed* to be introduced in the tropical colony.

## Childbirth practices through the lens of cultural and geographic tropes

“When the pains begin, send for the *dhai*. If the *dhai* chances to be wearing decent clothes, she will stop to change into the rags she keeps for the purpose infected and re – infected from the succession of diseased cases that have come into her practice. And so, at her dirtiest, a bearer of contagions, she shuts herself in with her victim.”^74^

While colonial medical discourse dwelt on tropical bodies and their ‘vulnerabilities’ due to supposed racial weaknesses, it was also fixated on the idea that certain cultural practices were causal factors in deteriorating maternal health. Medical journals reported on “filthy” childbirth practices and associated cultural norms, hinting that a ‘sanitary reform’ should be introduced as a part of the medicalisation of childbirth.^75^ The focus of this discussion largely remained on the training of the traditional midwives and making institutional childbirth accessible to Indian females.

Krishna Soman, in her paper on the politics of healthcare in Bengal, identified that traditionally the *dhais* among the Hindus belonged to the ‘untouchable’ caste/schedule caste.^76^ In this context, Anshu Malhotra has shown how in the late colonial period, the ‘space’ generally occupied by these ‘dirty’, low-caste women was politicised and appropriated by the elite and middle-class men and women. The attack on the caste and backwardness of the *dhais* was not only a colonial tool for introducing and establishing Western modernity. It was, as Malhotra argues, a ploy for gaining control over the space of women’s reproductive health.^77^

In 1927, Margaret Balfour, despite her condemnation of the Indian practices and illiteracy of the *dhais*, accepted that ‘there are several diseases of pregnancy for which the *dhais* are not responsible and which contribute largely to the fatal results.’^78^ Reporting a survey conducted at the different maternity hospitals in India, she emphasised: ‘The nature of the mortality is largely due to obscure diseases which will not be remedied merely by training midwives or opening maternity wards. Careful medical research is needed to discover the causes and means of prevention of these diseases. As at least two of them are nearly unknown in Britain… instruction in the diseases of pregnancy in tropical countries should form part of the curriculum of all schools of tropical medicine and research scholarships should be offered to help their investigation.’^79^

In her detailed article on early infant mortality in India, Balfour identified some ‘antenatal factors leading to weakness either of the germ cells or of the developing foetus or of both …and this may be connected with a maternal or paternal dietary deficiency.’^80^ Christine Thomson, in her survey on neo-natal deaths occurring in Indian hospitals showed how the chief cause of such deaths was ‘complication of labour’ which included ‘contracted pelvis, malpresentations, prolapsed of the cord, and dystocia from various causes including forceps delivery…ante partum hemorrhage when it occurs in India, takes the usual heavy toll of foetal and neo natal life’^81^

In the late colonial period, a book that dealt with cultural ideas on hygiene, sexual practices, maternal health and childbirth and related them to religious customs was the polemical *Mother India* (1927) by American journalist Katherine Mayo (1867–1940).^82^ The book was vigorously criticised by nationalist leaders. Gandhi remarked it to be a ‘drain inspector’s report’.^83^ It became a galvanising point for the nationalists^84^ but *Mother India*, nonetheless, made some important observations. Fearing the transmission of the ‘dangerous cultural practices’ from India to other parts of the world, Mayo reported that birthing chambers were horrors for the mothers because of the practices of the childbirth attendants or the *dhais*. Asha Nandkarni describes Mayo’s metonymic use of the term Mother India to denote the pathological body where the ceremonially ‘unclean’ parturient women could only come in contact with the unclean *dhais* with filthy habits who belonged to the untouchable class.^85^ Quoting Dr Marion A. Wylie, who was part of the MSF or ‘Doctors Without Borders’ group and wrote extensively about her medical experiences in India, Katherine Mayo reported that the *dhais* “preferred to extract the child by main force and the patient in such cases was badly torn, often into her bladder with the resulting large vesico-vaginal fistulae, so common in Indian women.”^86^

The following table gives a glimpse into the data (Table 3):

**Table 3.** Data collated from the Ceylon Administration Report, 1927, 36–7

Cause of death	Infantile and maternal mortality
Enteric fever	13
Advanced anchylostomiasis	34
Pneumonia	107
Puerperal septicaemia	16
Puerperal eclampsia	06
Placenta previa	10

In 1877, Dr P.S. Koomarsamy, native surgeon of Tanjore, observed that, unlike in Europe, dark- skinned women were peculiarly prone to puerperal convulsions, and this proclivity was due to the strange and injurious conditions of their confinement.^87^ In this context, he mentioned the role of ignorant midwives who often allowed patients to die in agony and pain but did not call for a doctor at the right time. About half a century later, Margaret Balfour (1929) reiterated similar concerns: ‘It is in childbirth that the full horror of the purdah system is seen, when women are allowed to die undelivered sooner than show themselves to a man’.^88^ However, in cases of protracted labour, the midwives were of little help, especially because of their lack of medical knowledge. Another Dr Mootosamy described the

… mischievous meddling of uneducated native midwives was attended with almost fatal results… enormous tension and swelling of the vulva and perineum, the dryness of the passages and the subsequent sloughing of the labia majora were clearly the outcome of the *midwife’s vigorous and ill – directed exertions.* ^89^

The contemporary Bengali and English medical journals like *Bamabodhini Patrika, Swasthya, Chikitsa Sammilani, Antahpur* and the *British Medical Journal*, as well as a few books and treatises, discussed the pain of parturient women. Annada Charan Kahstagir and Khiroda Prasad Chattopadhyay and others were regular columnists in these journals who wrote on female health issues, particularly on childbirth and child rearing. The ‘place of childbirth’, i.e. the *sutikagriha* was often identified as a place of noxious vapours that resulted in the death of the mother and child. The confinement rooms in most of the regions were small, close, damp and ill-ventilated, with closed doors and windows. Fumigation by burning charcoal was used to keep the rooms warm, but in the absence of any ventilation, the room would become a ‘hermetically sealed chamber’.^90^ Moreover, the unfortunate woman was literally starved for the first three days after delivery. The Parsees had the tradition of confinement for forty days, after which every piece of clothing was given to the sweepers or *bhangis.* In many cases, the sweepers used to sell them to poor Parsee women expecting their confinement, and thus, PF and other diseases were propagated throughout the community. Infections were inevitable as a pessary, consisting of a tampon of cloth dipped in brandy, was applied to the Parsee ladies for the first four days after delivery.^91^ In that particular state of exhaustion, induced by the shock of labour, the parturient women were especially liable to disease and when in such a condition she was deprived of food, pure air, light, and exposed to damp and cold, tetanus was the inevitable result.^92^ A report in the *Indian Medical Gazette* described that the traditional methods of the *dhais* in separating the umbilical cord with a bamboo stick picked up from the mud (unlike in Europe with a clean pair of scissors) was the obvious route for the tetanus bacillus to gain entry into the infant’s body through the navel. The insanitary ways of dressing the divided cord with cow dung ashes and covering it with burnt rag were deeply condemned by the Western doctors who argued incessantly for trained midwives.^93^

Katherine Mayo, in her book *Mother India*, gave a vivid account of the process of non-institutional childbirth in India, as facilitated by the *dhais.* She noted that ‘The little mother goes through a destructive pregnancy, ending in a confinement whose peculiar tortures will not be imagined unless in detail explained…the infant that survives the birth strain… bankrupt in bone stuff and vitality, often venereally poisoned, always predisposed to any malady that may be afloat…’^94^ Mayo described the experience of female doctors in India and their visits to Indian households during childbirth. She argued that girls suffered from their feeble and diseased ancestry, poor diet and the custom of early marriage, which rendered them too small-boned and diseased to be able to give birth normally to a child. She referred to Dr Vaughan’s report, which narrated a gruesome scene of childbirth in an Indian household: “That women … died of septicaemia contracted either from the dirty clothing which is saved from one confinement in the family to another (unwashed) or from the *dhai* who did her best in the absence of either hot water, soap, nail brush or disinfectants.”^95^

Dr K.O. Vaughan, who was a surgeon in the Zenana Hospital at Srinagar, reported regularly about her experiences and was quoted several times by Katherine Mayo in her book. In one instance, Dr Vaughan mentioned how such ghastly practices were common even in the households of educated Indians. She reported how an Indian medical man, who was in charge of a government centre for the training of *dhais* in modern midwifery, yielded to the pressure of his family and called an old-school *dhai* to attend his wife in confinement. Attended by the ‘dirty and ignorant’ *dhai*, the wife died of PF, and the child died at birth.^96^ Interestingly, Dr Vaughan mentioned that it was believed that PF was caused by *fresh air*, and the remaining air was vitiated by the presence of a charcoal fire burning in a pan.^97^

Agreeing with Mayo’s depiction of the deplorable condition of Indian women, M. Edith Craske in 1930 argued that these *dhais* were aware of their incompetence yet, even at the risk of the lives of the mother and child, they never sent for help.^98^ Though patients were reluctant to call for medical practitioners and trained *dhais*, especially in the non-institutional deliveries, lying–in hospitals were changing the nature of midwifery practice. Improvement in midwifery practice and knowledge of bacteriology were employed in dealing with problems of childbirth like sepsis, vaginal inflammation and PF.^99^ Medical practitioners engaged in the maternity wards of the lying-in hospitals emphasised that meticulous teaching was the only way to change childbirth conditions to ensure the safety and comfort of the patient. Use of aseptic gowns and wearing sterilised rubber gloves was prescribed for serious abdominal operations, along with detailed instructions to the midwifery assistant to remove the placenta and to encourage the contraction of uterus by the application of pressure immediately after delivery to prevent postpartum haemorrhage.^100^

In the 1920s and 30s, a number of books were published which dealt with the medical aspects of labour and childbirth, along with social practices and habits of childcare and maternal wellbeing. In 1922, *Hygiene for Health Visitors, School Nurses and Social Workers* was published by C.W. Hutt. It emphasised that a lack of sufficient knowledge of feeding, clothing, of personal and domestic hygiene was responsible for a lot of ill health and mortality amongst children, especially of the working classes. In reviewing the book, the *Indian Medical Gazette* specially mentioned a chapter which dealt with the care of infants and children and the role and duties of health visitors and school nurses in providing such care.^101^ Another book, written in the next year, *The Place of Version in Obstetrics*, by Dr Irving Potter, noted how ‘version’^102^ should be included in the syllabus of midwifery to decrease the mortality of both mothers and children.

With increased maternal mortality afflicted by puerperal diseases and disdainful parturient practices of the midwives leading to puerperal infections, knowledge of bacteriology was deemed to be adeemed to be a necessity in dealing with problems of childbirth like sepsis, vaginal inflammation and PF. However, Indian medical practitioners were at the same time increasingly insistent on the importance of prenatal and antenatal care, and the role of midwives/ childbirth attendants, as they were crucial in the prevention of the spread of bacteriological infections. It was in this context that the discussion on gynaecological health centred around the concept of tropicality as well as the colonial peculiarity of puerperal disease. Thus, the bacteriological understanding of the cause of maternal mortality used tropicality as a geographical and cultural trope to emphasise the colonial rule of difference.

Though in the foregoing discussion, the focus has remained on colonial India, this paper shall conclude by showing that colonial policies reflected some of the wider concerns of the global hygiene movement. That the modernisation of childbirth was at the centre of this global initiative, shall be evident from the following section. Bacteriological research was crucial to the prevention of maternal mortality, as was the role of birth attendants. While the former remained conspicuously absent in India, the late colonial period was marked by the adoption of specific colonial policies towards the latter. However, these policies could not evade their ‘colonial’ nature and continued to be marked by the racial and political prejudices of colonial administrators. The globally reinvigorated shift towards sanitation and hygiene as an integral part of female reproductive health was appropriated in India, both by colonial policy makers and their Indian counterparts. The passing of the Nursing and Midwifery Acts in the provinces,^103^ training of midwives and the insistence on institutional care of parturient women to maintain hygiene and sanitation reflected this global shift. The colonial critique of Indian cultural backwardness, unhygienic social practices and customs of unclean medical (and non-medical) agents was rephrased in a globally emerging modern language of sanitation and hygiene.

## Imperial hygiene and colonial policies: professionalisation of midwifery

One of the founding principles of the Dufferin Fund was to provide medical tuition for the training and teaching of nurses and midwives, as well as provision for trained nurses and midwives to care for women and children in hospitals and private houses. Until the end of the nineteenth century, it largely replicated the philanthropic model of Britain and depended on voluntary contributions.^104^ From different quarters, the 1880s and 1890s were marked by a clamour concerning the urgent need for women physicians. In the colonies, as Geraldine Forbes argues, British women played a crucial role at this time, entering the medical profession, ‘as it legitimized their professional goals and promised employment’.^105^

As Ambalika Guha has argued in her detailed work on the medicalisation of childbirth in India, the first step towards such policies was initiated by the inclusion of midwifery as an academic subject in the Calcutta Medical College, after the promulgation of the Medical Amendment Act of 1866. It stipulated that examination in midwifery be made compulsory along with medicine and surgery.^106^ While initiated through the modernising impulse and later promoted by a nationalist approach, such medicalisation drew heavily on Western medical science and technology.^107^ Guha argues that in the years post-1918, the value of maternal life as a factor determining the birth of healthy infants became entwined with eugenic discourse on the improvement of the race. Thus, the drive to professionalise midwifery emanated from the broader and globally relevant eugenic ideas. While Guha focuses on the ideas of nationalism and modernity in bringing out the Indian appropriation of the ideas of professionalising midwifery as a part of the medicalisation of childbirth, this paper attempts to trace the shift in the colonial policies and the role of the government in the process of professionalising of midwifery. Globally, traditional midwives were regarded as ignorant, dirty anachronisms, incapable of appreciating the need for cleanliness or of understanding the basic anatomical and physiological principles of the birth process. In 1934, Dr Feus, medical resident of the Nawab of Jamnagar, reported that he had opened a female hospital and recruited a ‘staff of nurses, the nucleus being four Indian women with certificates from the Bombay Nursing Association’. He opined that training the traditional *dhais* was a futile venture as ‘A woman of this type, slave for years of the dirty customs of her class, cannot be expected to throw aside the habits of a lifetime and imbibe the necessary knowledge of asepsis and midwifery of which she is at present totally ignorant, and to which she is by nature opposed. However earnest and sound the training may be, she would relapse to her old ways.’^108^ Rather, he argued in favour of training young Indian widows in courses of nursing and midwifery, as nursing in the prenatal and antenatal times was held to be equally important to prevent mortality as the process of childbirth itself. However, it was increasingly evident that doing away with the midwives entirely was an impossibility. Thus, in the next few decades, the professionalisation of midwifery was carried out as an administrative policy to negotiate between domestic hygiene and medical institutionalisation.

In the context of bacteriological research, one can see a reinvigorated discussion on the role of birth attendants/*dhais*/midwives and nurses. Such discussions on cultural practices in tropical colonies, in antenatal and postnatal healthcare, occurred with a shifting focus, based on the language of ‘hygiene’. Theoretical exploration of the links between public health policies and assessments of ‘civic virtue’ has been a repeated theme of academic endeavours in the last two decades. In this context, the project of ‘Imperial Hygiene’, as coined by Alison Bashford, was shaped through the ‘development by sanitation…colonising by the means of the known laws of cleanliness’.^109^ Deana Heath, in the context of regulating obscenity in colonial India, discusses how imperial hygiene marked the shift from ‘moral question to a medical and racial concern’ in the late nineteenth and early twentieth century.^110^ However, the arguments are mostly based on medical–administrative knowledge and its effects on policies in relation to diseases like plague, cholera, smallpox, tuberculosis and other epidemic diseases, which directly affected the health of the colonial (military and European population) power. Keeping with the emergent theoretical frame of global history of tropical medicine,^111^ this article has evaluated how ideas of imperial hygiene defined colonial maternal health policies in the late colonial period by locating the agents of ‘racial hygiene’ in their different social, institutional and administrative settings.

The ideas shaping imperial hygiene can be traced back to the late nineteenth century. It was during the post-Crimean War and in the context of European epidemics of PFs, that Florence Nightingale emphasised the role of every woman as a nurse, as she was opposed to the Victorian myth that considered home to be a confinement or a hotbed of disease.^112^ Thus, as an effective means to eliminate diseases, she recommended separation between the hospitals and the lying-in wards, and suggested that only trained midwives attached to the lying in wards could attend births to promote hygiene and prevent contagion.^113^ It has been argued by scholars like Mary Poovey that Nightingale’s idea of ‘care and hygiene’ was conspicuously supporting Britain’s imperial designs in India.^114^

Such ideas gained momentum in the second and third decades of the twentieth century. Narita Ruichi showed how in early twentieth-century Japan, hygiene was crucial to the role of women – regular columns were written informing on menstrual hygiene, childbirth practices, breastfeeding, personal hygiene and childcare. He discussed how for puerperal, or childbed, fever, obstetrician Iba Hideaki recommended ‘complete disinfection’ as a preventive measure, avoiding old rags and using only sterile gauze or cotton.^115^ In the U.S., the Promotion of the Welfare and Hygiene of Maternity and Infancy Act, more commonly known as the Sheppard–Towner Act (1921), recognised the importance of factors like poverty, lack of prenatal care, unsanitary living conditions and contaminated milk and water in contributing towards high maternal and infant mortality. While the incidence of puerperal sepsis could be reduced by observing aseptic techniques, other potentially fatal diseases like toxaemia, pelvic disproportion, malpresentation and haemorrhage could be prevented by conscientious prenatal care and skilled practitioners, coined by J.W. Ballantyne as a ‘new midwifery’.^116^ In 1925, Mary Breckinridge founded the Frontier Nursing System, where nurse–midwives could participate in prenatal and antenatal care as well as work as health educators, instructing families in the basic principles of sanitation, nutrition and childcare. Breckenridge was impressed by the work of the French and British nurse–midwives she wrote in her autobiography that ‘it grew upon me that nurse midwifery was the logical response to the needs of the young child rural America.’^117^

In India, it was the anti-quarantine issue that sparked efforts focusing on hygiene and sanitation. In terms of female health, language remained focused on training midwives and health officials about the importance of hygiene and its preventive role in the spread of puerperal diseases.^118^ The most important impact of this global movement on hygiene and female health was manifest in the role of the Rockefeller Foundation in the establishment of the All India Institute of Hygiene and Public Health in 1932, which had a reinvigorated prioritisation of hygiene in prenatal and antenatal healthcare. This institution, located in the vicinity of Calcutta Medical College, had continuous intellectual and medical interactions with the latter and for both the institutes, hygiene, diet, and sanitation became an integral part of teaching and practice.

Indian provinces also passed several pieces of legislation which had the primary aim to register *trained* nurses, midwives and *dhais* and to ‘provide for the registration of nurses and midwives in order to protect both the public and properly qualified practitioners from the very large number of incompetent and untrained women, practising as nurses’.^119^ With the official prohibition of the practice of the untrained *dhais*, the provinces quite openly accepted the Western training in female healthcare through legislative means. The provinces of Madras (1926), Bihar and Orissa (1935), Bengal (1934), and United Provinces (1934) were unanimous in accepting trained nurses and midwives registered with the Council to be the norm of their province. According to the Acts, a Council was formed for the registration of the nurses and no one without registration could be appointed as ‘matron, superintendent of nursing, sister, staff nurse, nurse, mid wife, assistant midwife or health visitor’ in any dispensary, hospital, asylum, infirmary, lying-in hospital, or maternity and child welfare centre which were supported wholly or partially from state funds.^120^ In 1934, Dr Orkney, a practitioner at the All India Institute of Hygiene and Public Health in Calcutta reiterated the importance of the diploma in Maternity and Child Welfare and argued that the purpose of visits of midwives during puerperium was to attend to the general cleanliness and comfort of the mother, the establishment and management of breastfeeding, the care and cleanliness of the baby and the general hygiene of the surrounding.

## Conclusion

I have argued that bacteriological research in relation to female health proved crucial to health policies in colonial India. Though maternal mortality had been a concern for the longest time, PF could be diagnosed, addressed and prevented to a large extent with the coming of germ theories and bacteriological research. However, as other historians have also demonstrated, colonial policies failed to remedy the lack of bacteriological research carried out in India in relation to female health and maternal mortality. This paper has identified how medical practitioners in India were mainly trained in Europe and proved to be the channels through which such scientific ideas moved to India. Marking the coloniality of such colonial medical discourse, the ideas of ‘tropicality’ defined ideas about the female body and pathological ideas related to childbirth. Historians of colonial health have delved deeply into the factors promoting bacteriological research in India, focusing on the epidemic diseases of cholera, tuberculosis, plague and kala azar. The focus was on preventive medicines, and vaccination was one of the primary research agendas, especially in the Haffkine Institutes. This relegated the relative importance of female health in terms of research. But in this connection, colonial trade policies, especially in relation to cholera, was in the midst of conflicting international opinions about quarantine and sanitation, which, as this paper has argued, had a significant bearing on the colonial discourse on hygiene and sanitation, especially in relation to the institutionalisation and professionalisation of midwifery and childbirth attendants. British policy on sanitation created a ‘stasis’ in research on one hand, but discussion about cleanliness, hygiene, prenatal and postnatal care, and maternal and infant care on the other hand initiated a modernised discourse on hygiene and sanitation related to female health. The most important impact was manifest in the professionalisation of childbirth attendants, marking a shift to nursing–midwifery.

## Footnotes

1 Pratik Chakraborty, *Bacteriology in British India* (New York: University of Rochester Press, 2012), 61.

2
*Ibid.*, 62.

3 Anne Hardy, ‘Cholera, Quarantine and the English Preventive System’, *Medical History*, 37 (1993), 250–269.

4 Danielle Gallardo-Albarran, *The Global Sanitary Revolution in Historical Perspective*, EHES Working Paper, No. 247, January, 2024, https://www.econstor.eu/bitstream/10419/298572/1/1878616552.pdf

5 Supriya Guha, ‘From Dias to Doctors: the Medicalisation of Childbirth in Colonial India’ in L. Lingam (ed.), *Understanding Women’s Health Issues: A Reader* (New Delhi: Kali, 1998). 228–235, http://www.womenstudies.in/elib/others/ot_from_dias.pdf; Supriya Saha, Midwifery, Childbirth and Breastfeeding Advice in Colonial Bengal, in Lata Singh and Shashank Shekhar Sinha (ed.), *Gender in Modern India: History, Culture and Marginality* (Oxford University Press, 2024), 203–222.

6 Christine Hallett, ‘The Attempt to Understand Puerperal Fever in the Eighteenth and Early Nineteenth Centuries: The Influence of Inflammation Theory’, *Medical History*, 49, 1 (2005 January), 1–28; Christine DeLacy, Puerperal Fever in Eighteenth-century Britain, *Bulletin of the History of Medicine*, 63, 4 (1989), 521–556; Thomas Denman, *Essays on the Puerperal Fever and Puerperal Convulsions*, (London: J Walter, 1768), 8–10; William Fordyce, *A New Inquiry into the Causes, Symptoms and Cure of Putrid and Inflammatory Fevers* (London, 1777), 12–14.

7 Irvine Loudon, *The Tragedy of Childbed Fever* (Oxford: Oxford University Press, 2000), 6.

8 G.W. Lowis, ‘Epidemiology of Puerperal Fever: The Contributions of Alexander Gordon’, *Medical History*, 37 (1993), 399–410.

9 Alexander Gordon, *A Treatise on the Epidemic Puerperal Fever of Aberdeen*, (London: G.G. and J. Robinson, 1795). R.M. Woodbury, *Infant Mortality and Its Causes: With an Appendix on the Trend of Maternal Mortality Rates in the United States*, (Baltimore: The Williams and Wilkins Company, 1926). I.M. Gould, ‘Alexander Gordon, Puerperal Sepsis, and Modern Theories of Infection Control. Semmelweis in Perspective’, *Lancet Infect Dis.* 2010 Apr;10(4):275–8.

10 Hallett, *op. cit*. (note 6), 5.

11 G.P. Parsons, ‘The British Medical Profession and the Contagion Theory: Puerperal Fever as a Case Study, 1830–1860’, *Medical History*, 22 (1978), 138–150.

12 Irvine Loudon, ‘The Cause and Prevention of Puerperal Sepsis’, *Journal of the Royal Society of Medicine*, 93 (2000). https://journals.sagepub.com/doi/pdf/10.1177/014107680009300720

13 R.C. Lancefield and R. Hare, ‘The Serological Differentiation of Pathogenic and Non-pathogenic Strains of Hemolytici Streptococci’, Journal of Experimental Medicine, 61 (1935), 335–349. doi: 10.1084/jem.61.3.335.

14 J. Baker and F. F. Barrett, ‘Transmission of Group B Streptococci among Parturient Women and Their Neonates’, *Journal of Pediatrics*, 83 (1973), 919–925. D. I. Lansing, W. R. Penman and D. J. Davis, ‘Puerperal Fever and the Group B BETA Hemolytic Streptococcus’, *Bulletin of the History of Medicine*, 57, 1 (1983), 73. M.J. Patterson and A.E.B. Hafretz, ‘Group B Beta Hemolytic Streptococcus in Human Disease’, *Bacteriological Revolution*, 40 (1976), 774–778.

15 Ronald Hare, ‘Puerperal Sepsis and Its Prevention, Part I’, *Canadian Public Health Journal*, 28, 11 (1937), 554–63.

16 D. Arnold, ‘Cholera and Colonialism in British India’, *Past and Present*, 113 (November 1986), 118–151. B. Bulmus, *Plague and Quarantines in the Colonial Era, Plague, Quarantine and Geopolitics in the Ottoman Empire* (Edinburgh University Press, 2012). S.N.Dey, *Cholera: Its Pathology and Pathogenesis*, (Edinburgh and London: Oliver & Boyd. 1961). W.F. Bynum, *Science and the Practice of Medicine in the Nineteenth Century* (Cambridge University Press, 1994). Aparna Basu, ‘The Indian Response to Scientific and Technical Education in the Colonial Era, 1820–1920’, in Deepak Kumar (ed.), *Science and Empire: Essays in Indian Context (1700–1947)* (Delhi: Anamika Prakashan, 1991), 126–38. R. Ramasubban, *Public Health and Medical Research in India: The Origins under the Impact of British Colonial Policy*, (Stockholm: Sarec, 1982).

17 P. Chakrabarti, ‘Beasts of Burden: Animals and Laboratory Research in Colonial India’, *History of Science*, 48, 2 (2010), 125–152.

18

John Mathew , ‘Ronald Ross to U N Brahmachari: Medical Research in Colonial India’, Indian Journal of History of Science, 53 (2018), 115–22.

19 I. Klein, ‘Plague, Policy and Popular Unrest in British India’, *Modern Asian Studies*, 22 (1988), 723–755

20 David Arnold, ‘Science, Technology and Medicine in Colonial India’, in *The New Cambridge History of India*, Vol. 3 (Cambridge University Press, 2000), 141–7.

21 Margaret I. Balfour and Ruth Young, *The Work of Medical Women in India* (London: Oxford University Press, 1929), 125.

22 Ramasubban, *op. cit*. (note 16).

23 Mridula Ramanna, ‘Maternal Health in Early Twentieth Century Bombay’, *Economic and Political Weekly*, 42, 2 (2007), 138–44.

24 David Arnold, ‘Colonial Medicine in Transition: Medical Research in India, 1910 – 1947’, *South Asia Research*, 14, 1 (1994), 20.

25

David Arnold , Colonizing the Body: State Medicine and Epidemic Disease in Nineteenth Century India (Berkeley, CA: University of California Press, 1993), 198 .

26 5 Curzon to J. Broadrick (the Secretary of State), January 28, 1904, British Library, London, India Office Records and Private Papers, Curzon Papers, IOL, Mss. Eur F.111/163.

27 E.W.C. Bradfield, ‘Medical Education’, *Indian Medical Gazette* (1928), 90.

28 The cholera conferences were held as part of International Sanitary Conferences. Between 1851 and 1938, 14 conferences were held to discuss the need for and ways to control the spread of diseases such as cholera. While most European governments were in favour of exploring quarantine as a measure, British medical and diplomatic delegates continued to protest against it in all the conferences (https://iris.who.int/bitstream/handle/10665/62873/14549_eng.pdf).

29

Sheldon Watts , ‘From Rapid Change to Stasis: Official Responses to Cholera in British Ruled India and Egypt, 1860 – 1921’, Journal of World History, 12, 2 (2001), 321–7420039497 10.1353/jwh.2001.0047.

30 Deepak Kumar, ‘Racial Discrimination and Science in Nineteenth Century India’, *Proceedings of the Indian History Congress*, 41 (1982), 52–74 (750).

31 ‘Medical Research in India’, *British Medical Journal*, March (1917), 427–8.

32 Margaret Balfour, ‘Maternal Mortality in Childbirth in India’, *Indian Medical Gazette*, 62, 11 (November 1927), 646–647.

33 David Arnold, *op. cit.* (note 25), 18.

34 Kiple and Ornelas, ‘Race, War and Tropical Medicine in Eighteenth Century Caribbean’, in D. Arnold (ed.), *Warm Climate and Western Medicine: The Emergence of Tropical Medicine 1500–1900* (Brill, 1996); L. Wilkinson, ‘Rinderpest and Mainstream Infectious Disease Concepts in the Eighteenth Century’, *Medical History*, 28 (1984), 129–150; W. Anderson, *Colonial Pathologies: American Tropical Medicine, Race, and Hygiene in the Philippines*, (Durham and London: Duke University Press, 2006).

35 W. Anderson, *The Cultivation of Whiteness: Science, Health and Racial Destiny in Australia* (Melbourne University Press, 2002), 73.

36 Warwick Anderson, ‘Disease, Race and Empire’, *Bulletin of the History of Medicine*, 70, 1 (1996), 62–7 (63).

37
*Ibid.*, 65.

38 W. Dissanayake, ‘Exotic Other: Western Representation of India in English Literature and Film’, *World Englishers*, 5, 2–3 (1986), 177–87.

39 N. Kokayeff, ‘Dying to Be Discovered: Miasma vs Germ Theory’, *ESSAI*, 10 (2012), 68–72; T. Koch, ‘Mapping the Miasma: Air, Health and Place in Early Medical Mapping’, *Cartographic Perspectives*, 4, 52 (2005), 4–27; M. Karamanou, ‘From Miasmas to Germs: A Historical Approach to Theories of Infectious Disease Transmission’, *Infezioni in Medicina,* 20, 1 (2012), 52–56; B. Cleetus, ‘Tropics of Disease: Epidemics in Colonial India’, *Economic and Political Weekly (Engage)*, 55, 21 (23 May 2020).

40 Chakraborty, *op. cit.* (note 1), 4.

41
*Ibid.*, 5.

42 Arnold, *op. cit.* (note 24), 10–35.

43 Chakraborty, *op. cit.* (note 1), 61.

44

Partha Chatterjee , The Nation and Its Fragments: Colonial and Post Colonial Histories (Princeton: Princeton University Press, 1993), 9.

45 Maneesha Lal, ‘The Politics of Gender and Medicine in Colonial India: The Countess of Dufferin’s Fund, 1885–1888’, *Bulletin of the History of Medicine*, 68, 1 (1994), 29–66.

46 Geraldine Forbes, ‘Medical Careers and Health Care for Indian Women: Patterns of Control’, *Women’s History Review*, 3, 4 (1994), 517.

47 The fund was created by Queen Victoria to provide health services to Indian women. But the fund relied on trained Western women in providing services rather than on the training of native women.

48 M. Lal, ‘The Politics of Gender and Medicine in Colonial India: The Countess of Defferin’s Fund, 1885–1888’, *Bulletin of the History of Medicine*, 68, 1 (1994), 29–66.

49 G.R. Rao and A.T. Roy, ‘Mercurochrome – 220 Soluble in Leprosy Work’, *Indian Medical Gazette*, 67 (1932), 125.

50 Carmichael Medical College Hospital, *Annual Report*, 1927, 61.

51 ‘Dai’ or midwives have been also spelled as ‘dhai’ in the official documents and primary documents. I have followed the orthography of the source.

52 ‘Infantile Mortality in India’, *Indian Medical Gazette*, 42 (1907), 302–3.

53 ‘Notes on a Case of Puerperal Eclampsia Treated by Hypodermic Injection of Morphia’, *Indian Medical Gazette*, February (1904), 60.

54 Lucy Wills and M.M. Mehta, ‘Studies in Pernicious Anaemia in Pregnancy: Preliminary Report’, *Indian Journal of Medical Research*, 17 (1930), 777–92.

55 V.B. Green Armytage and P.C. Dutta, *A Textbook of Midwifery in the Tropics* (Calcutta: The Book Company Limited, 1933).

56
*Ibid.*, 34.

57 V.B. Green-Armytage, ‘Tropical Gynecology and Obstetrics’, *Indian Medical Gazette*, 68, 3 (1933), 151–152.

58 Green Armytage and Dutta, *op. cit.* (note 55), 31.

59 Guha, ‘The Nature of Woman: Medical Ideas in Colonial Bengal’, *Indian Journal of Gender Studies*, 3, 1 (1996), 29.

60 Ishita Pande, *Sex, Law and the Politics of Age: Child Marriage in India, 1891 – 1937* (Cambridge: Cambridge University Press, 2020). See also, Ashwini Tambe , Defining Girlhood in India: A Transnational History of Sexual Maturity Laws (Chicago: University of Illinois Press, 2019).

61 Green-Armytage, op. cit. (note 57), 35–6.

62
*Ibid.*, 35.

63 Koi, magur and singee are local varieties of fish. Khoi is popped rice, muri is puffed rice, chira is flattened rice, atta is wheat flour and suji is semolina. Green-Armytage, *op. cit*. (note 57), 38.

64 Green Armytage and Dutta, *op. cit.* (note 55), 33.

65 Chakraborty, *op. cit.* (note 1), 3.

66 Green-Armytage and Dutta, *op. cit.* (note 55), 87–8.

67 ‘The Treatment of Puerperal Sepsis’, *Indian Medical Gazette*, April 1927.

68 K.N. Das, ‘Obstetrics and Gynaecology’, *Indian Medical Gazette*, 36, 5 (1901), 194–5.

69
*Ibid*.

70 Kedarnath Das, ‘The Bengal Forceps’, *Indian Medical Gazette*, 58 (1923), 22–7.

71 Guha, *op. cit.* (note 59), 29.

72 Green-Armytage and Dutta, *op. cit.* (note 55), 30.

73 Carmichael Medical College Hospital, *Annual Report, 1927*, 62.

74 Katherine Mayo, *Mother India* (New York: Blue Ribbon Books, 1928), 93.

75 C.J. Thompson, *Report of an Enquiry into Stillbirth in India* (New Delhi: Lady Irwin Research Fund, 1930).

76 Krishna Soman, ‘Women, Medicine and the Politics of Gender: Institution of Traditional Midwives in Twentieth Century Bengal’, IDSK, *Occasional Paper*, 2011, 26.

77

Anshu Malhotra , ‘Of Dais and Midwives: Middle Class Interventions in the Management of Women’s Reproductive Health: A Study From Colonial Punjab’, Indian Journal of Gender Studies, 10 (2003), 230 .

78 Margaret Balfour, ‘Maternal Mortality in Childbirth in India’, *Indian Medical Gazette*, 62 (1927), 646.

79
*Ibid.*, 655.

80 Margaret Balfour, ‘Early Infant Mortality in India With Special Reference to Premature Birth’, *Indian Medical Gazette*, (1930), 630–7, see 636.

81 Christine Thomson, ‘Still Birth and Neo Natal Death in India: A Preliminary Inquiry’, *IJMR* (1931), 491–2.

82 The book was an elaborate critique of Indian culture and narrated the deplorable conditions of Indian women. K. Mayo, *Mother India* (New York: Blue Ribbon Books, 1927).

83 M.K. Gandhi, ‘A Drain Inspector’s Report’, https://www.gandhipedia150.in/static/data/highlighted_pdfs_output/tagore_volume34_book_452.pdf.

84 S. Mukherjee, ‘Katherine Mayo’s Mother India and Its Critique by the Diaspora’, *Proceedings of the Indian History Congress*, 78 (2017), 820–827.

85

Asha Nandkarni , ‘Word Menace: National Reproduction and Public Health in Katherine Mayo’s Mother India’, American Quarterly, 60, 3 (2008), 818 .

86 Mayo, *op. cit.* (note 74), 94.

87 ‘Contribution to the Practice of Midwifery’, *Indian Medical Gazette*, November (1877), 290–1.

88 Balfour and Young, *op. cit*. (note 21), 3.

89 ‘Contributions to the Practice of Midwifery’, *Indian Medical Gazette*, July 1 (1880), 186–7.

90 ‘The Native Methods of Treating Lying – In Women in India’, *Indian Medical Gazette*, November (1885), 350–3.

91
*Ibid.*

92 ‘Contribution to the Practice of Midwifery’, *Indian Medical Gazette*, November (1877), 290–1.

93 ‘Infantile Mortality in India’, *Indian Medical Gazette*, August (1907), 302–3.

94 Mayo, *op. cit.* (note 74), 38.

95
*Ibid.*, 100.

96
*Ibid.*, 101.

97
*Ibid.*, 99.

98 M. Edith Craske, *Sister India: One Solution for the Problems of Mother India* (London: The Religious Tract Society, 1930), 75.

99 ‘Hereditary Midwives in India’, *British Medical Journal*, 2, 2118 (1901), 294.

100 A. Guha, *Colonial Modernities: Midwifery in Bengal*, 1860–1947 (Calcutta: Routledge India, 2018), 65–68.

101 ‘Review’, *Indian Medical Gazette*, March (1922), 116.

102 ‘Version’ is the procedure to turn the foetus from side–lying or transverse position to the head–down or vertex position.

103 The Madras Nurses and Midwives Act, 1926; The United Provinces Nurses, Midwives, Assistant Midwives, Auxiliary Nurse-Midwives and Health Visitors Registration Act, 1934; The Bengal Nurses Act, 1934; and the Bihar and Orissa Nurses Registration Act, 1935.

104 Lal, *op. cit.* (note 45), 37.

105 Forbes, *op. cit.* (note 46), 515–30.

106 Guha, *op. cit*. (note 59), 63.

107 Ambalika Guha, ‘Modernising Childbirth in Colonial India: A History of Institutionalisation and Professionalisation of Midwifery, c. 1860 – 1947’ (unpublished PhD thesis: Victoria University of Wellington, 2015), 5.

108 ‘Obstetrics in India’, *BMJ*, December (1934), 1062–4.

109 A. Bashford, *Imperial Hygiene: A critical History of Colonialism, Nationalism and Public Health* (London: Palgrave Macmillan, 2004), 1.

110 Deana Heath, ‘Sanitizing Modernity: Imperial Hygiene, Obscenity, and Moral Regulation in Colonial India’, in Saurabh Dube (ed.) *Enchantments of Modernity: Empire, Nation, Globalisation* (New Delhi: Routledge, 2009), 117.

111 Lioba A. Hirsch, and Rebecca Martin, *LSHTM and Colonialism: A Report on the Colonial History of the London School of Hygiene & Tropical Medicine (1899–c.1960). Project Report* (London: London School of Hygiene & Tropical Medicine, 2022).

112 L. Macdonald, ‘Florence Nightingale and the Early Origins of Evidence based Nursing’, *Evidence Based Nursing*, 4, 3 (2001), 68 –69.

113 Peter Dunn, ‘Florence Nightingale: Maternal Mortality and the Training of Midwives’, *Archives of Disease in Childhood – Fetal and Neo Natal Edition*, (1996), 218–20.

114 Mary Poovey, ‘A Housewifely Woman: the Social Construction of Florence Nightingale’, in *Uneven Developments, the Ideological Work of Gender in Mid-Victorian England* (Chicago: University of Chicago Press, 1988), 164–98.

115

Narita Ruichi and Gretchen Jones , ‘Women and Views of Women within the Changing Hygiene Conditions of Late Nineteenth- and Early Twentieth-Century Japan’, U.S.-Japan Women’s Journal. English Supplement, 1995, 8 (1995), 64–86 .

116 ‘The New Midwifery’, *BMJ*, April (1923), 618.

117 Nancy Schrom Dye, ‘Mary Beckenridge, the Frontier Nursing Service and the Introduction of Nurse Midwifery in the United States’, *Bulletin of the History of Medicine*, 57 (1983), 481.

118 ‘Hygiene and Sanitation in India’, *BMJ*, June (1914), 1317.

119 Government of India, Home Department, Judicial Branch, File No. 1248/33, 1933.

120 Government of India, Home Department, Judicial Branch, File Number 766/33, 1934.

